# Hospital and Institutional News

**Published:** 1912-04-06

**Authors:** 


### HOSPITAL AND INSTITUTIONAL NEWS.

### OUR SPRING SPECIAL NUMBER.

On April 27, the last Saturday of the present month, The Hospital will publish its Spring Special Number. This year we have been so much impressed with the importance to every institution of economical management in the stewards' and kitchen departments, which is illustrated so forcibly in our article on "The Comparison of Prices Paid by London Hospitals for their Food Supplies" this week, that we have decided to make the Modern Institution Kitchen the main feature of this special issue. The subject will be treated exhaustively in a series of specially illustrated and technical articles. In this way the Spring Number will be of value to all interested in the economical management of voluntary, Poor Law, and public institutions, the special administrative economies of which will be available for comparison.

### SIR ALMROTH WRIGHT'S LETTER TO A LAY NEWSPAPER.

Whatever may have been the reasons which led Sir Almroth Wright to put forward his personal opinions on feminine psychology in the guise of a scientific explanation of certain actions by militant women suffragists, no one can deny that a lay newspaper was not the place for the ventilation of such opinions. The correspondence which his recent letter in the *Times* for the most part has excited, has been, and in the majority of cases necessarily must be, of no scientific value. The whole subject therefore is dragged before those, with few exceptions, incompetent to discuss it, with no good result to science or to public opinion. Sir Douglas Powell's view that the experiences of the consulting-room should not be aired in lay newspapers is one with which the medical profession will agree, and when it is added that a prominent anti-women-suffrage society is reported to be circulating the letter as a political tract it will be admitted that the limit of illogical folly has been reached. Yet results of this kind can only be expected when a matter of scientific and professional interest is exploited in the market-place, beyond the pale of scientific discussion.

### THE MORAL OF THE DEBATE.

What may well be urged is that when great subjects of national importance involving sex, such as woman suffrage and divorce, come in for responsible discussion or inquiry, fundamental considerations should not be left to amateurs for treatment. It appears to be consistently forgotten or ignored that in this country we have an authority on sex who is one of the few English writers—and one of the fewer English informative writers—in possession of a large Continental and American audience. Mr. Havelock Ellis (happily a medical man) has devoted many years, much culture, and the ability of a born student, to the investigation of sex psychology. Yet neither his work nor even his name receives mention on occasions like the above. The persons listened to are, as we have seen, well-spoken barristers, eminent clinicians, general medical publicists, and, as lately, famous pathologists. But the man whose appreciation of these matters is to theirs as sunlight to moonshine, seems to be reckoned a nobody. It is one of those little national traits of ours that arouse, rather inconveniently, the German's heavy mirth. Cynics might say that this expert is suppressed as being likely to prove an inconvenient witness. But even leaving unchallenged such a pessimistic view of official morality, there is no reason in this argument, as all may find who care to read, to study, and, let us hope, to quote and acknowledge, the author named. In that case the trend of events will surely falsify Lord Robert Cecil's remark that although he has every respect for a distinguished man of science as such, yet there is hardly anybody he would not sooner take as a guide in practical politics; and one makes this prediction without any pronouncement for or against the subject of last week's debate. The point endeavour has been here made to elaborate is that when medical counsel of any kind has to be taken, that counsel should be the best available.

### THE VACANT CORONERSHIP.

The London County Council are about to advertise for a successor to the late Mr. J. Troutbeck, coroner for the South-Western district. Mr. Troutbeck was also coroner for the Westminster City and Liberty, and for the Duchy of Lancaster Liberty (Savoy portion), and his successor will also occupy those positions. The Dean and Chapter of Westminster have the right of appointment of the coroner for the Westminster City and Liberty, but they agree with the County Council's suggestion that the two offices should be held by the same man. The Chancellor of the Duchy of Lancaster likewise is prepared to appoint the selected candidate to be coroner for the Duchy of Lancaster Liberty (Savoy portion). The aggregate salary for the post is £1,150. Whoever is appointed we may hope with some confidence that the post will be given to a medical man. The day has gone by for assigning the post of coroner to a lawyer who is not a qualified practitioner.

### PRINCESS CHRISTIAN AND THE "QUINTON POLYCLINIC."

Few members of the Royal Family have carried out its traditions in respect of philanthropic work—especially where hospitals are concerned—in a more genuine spirit of sympathy than Princess Christian. It is, doubtless, this goodness of heart which prompted her Royal Highness to accept an invitation to be present at a meeting held last week of the subscribers and supporters of the "Quinton Polyclinic," and to allow her name to be added to the list of patronesses of the institution. Medical opinion in London, however, refuses to take the "Quinton Polyclinic" seriously; nor has any adequate answer ever been given to the pertinent criticisms directed against it, both in these columns and in those of other professional journals. Her Royal Highness was presumably unaware of this; but those who advised her to accept the invitation have incurred a heavy responsibility.

### CARDIFF MENTAL HOSPITAL AND A TRADE-UNION.

The local branch of the National Asylum Workers' Union recently forwarded a petition to the Lord Mayor of Cardiff complaining of the working conditions and the quality of the food, and calling for an inquiry. Moreover, one of the attendants, who, it was stated, had previously been reprimanded for a breach of the rules, for another offence had been given a month's notice. In consequence of this the Cardiff Trades and Labour Council desired an interview with the visiting committee, and urged that the attendant had been discharged because he was a trade-unionist. Alderman Morgan Thomas stated at a meeting of the visiting committee last week that the rules of the Lunacy Board, which were incorporated in their own rules, laid down that all complaints should come through the officers and the medical attendant to the visiting committee. He added that if discipline were to be maintained no outside interference in the management could be tolerated. Dr. Goodall, the medical superintendent, said that so far from the food being inferior a special official presided over each meal, and that his authority had been passed over in the petition to the Lord Mayor. Dr. Goodall's action was then confirmed, and the visiting committee decided to refuse to meet a deputation of the Trades Council. The whole affair, however, has an interest for all grades of mental hospital workers for that it supplies an example of the way in which an indiscreet official may cause endless difficulties.

### REPATRIATING A MENTAL HOSPITAL PATIENT.

The Mental Hospitals Committee of the London County Council proposes to spend £100 on the return to British Guiana of a patient in Banstead Mental Hospital who is a native of that colony. The committee states that the patient, who is a young woman, was admitted to Banstead in February 1911 only two months after her first arrival in this country. As she had no settlement in England and was without means, she has become chargeable to the County of London. The committee considers it desirable, both for her own sake and also with a view to relieve the county rate on which otherwise she might be a charge for many years, that she should be sent back to the colony in which she was born and where her relatives still reside. The officer administering the government of British Guiana has reported that if arrangements can be made for her passage back without expense to the colony there will be no difficulty in receiving her into the mental hospital there as an ordinary pauper patient. The Mental Hospitals Committee, however, has no fund at its disposal from which the expenses of repatriation could be defrayed, and without special authorisation these expenses could not be defrayed from the county rate. The total cost, including the expenses of an escort, should not exceed £100. The Local Government Board has now given the necessary sanction under the Local Authorities (Expenses) Act, 1887, to expend a sum not exceeding £100 for the purpose.

### VALUABLE GIFT TO THE SALOP INFIRMARY.

Two memorials have been unveiled to commemorate the services rendered to the Salop Infirmary by the late Chairman of the Board of Directors, Mr. James Cavan, of Eaton Mascott Hall. The first was the dedication of a brass tablet in the chapel of the Infirmary, bearing the following inscription: "In Memory of James Cavan, 15 years a director of this Institution, who died while Chairman of the Board, 25 April, 1911. This tablet was placed in the Chapel by the Treasurer and friends." After the short dedication service the treasurer, Mr. Hillyar Chapman, uncovered a handsome mahogany book-case containing between 500 and 600 volumes. It is surmounted by a brass plate bearing the words: "This library was presented to the Salop Infirmary in memory of James Cavan, late Chairman of the Board, by the Treasurer and friends. 1912." Mr. Alfred Mansell, deputy chairman, expressed the pleasure with which he, on behalf of the board of directors, accepted the library as a tangible memento of the late Mr. Cavan, who was for many years a director of the Infirmary and latterly its chairman. Mr. Cavan was a most generous supporter of the institution, having contributed in the last few years £1,000 to its funds. Mr. Mansell alluded to his well-known kindness and sympathy, and said he felt the subscribers were doing what he would have wished in providing a library for the comfort and pleasure of the inmates of the Infirmary. There is no doubt that a library of good and readable literature is a valuable adjunct to any hospital, whether from the point of view of the patients or of the resident staff. It is moreover one that is not as common as it ought to be, and the donors of this library deserve therefore thanks for their originality as well as for their generous intention.

### ROYAL OPENING OF NEW BUILDINGS AT THE MIDDLESEX HOSPITAL.

The Barnato-Joel cancer charity in connection with the Middlesex Hospital was opened last week by her Majesty the Queen. The hospital's president, the Duke of Northumberland, received her Majesty, who was conducted by Mr. F. Clare Melhado to the board-room, where a number of presentations were made. Among these were the five trustees, Lord Cheylesmore, Mr. J. B. Joel, Mr. S. B. Joel, Sir John Purcell, and Mr. A. G. Asher. The electrical department, the *x*-ray room, and the out-patient department were visited in turn, and as most of these had already begun work for some days, the Queen was able to see them in their activity. Countess Gleichen's bust of Mr. H. I. Barnato is in the centre of the ground floor, and the nurses' quarters and some of the wards were also inspected.

### THE BUILDINGS DESCRIBED.

It should be remembered that the £250,000 left by the late Mr. Harry Barnato in memory of his brother, Mr. Barney Barnato, has been used to provide a complete cancer hospital. The beds number forty-three, the nurses' home has accommodation for fifty-nine, and there are bedrooms for twenty servants. Apart from this there is a theatre, a research department, and an electrical department. Mr. Edwin T. Hall is the architect, and Mr. J. Holliday the builder. The foundation stone was laid in July 1910.

### TRANSFERRING PATIENTS AT WIMBLEDON.

At the annual general meeting of subscribers and donors to the South Wimbledon, Merton and District Cottage Hospital, held last week at the hospital's new building in Kingston Road, Merton, the chairman, Alderman Summerhays, S.C.C., and Mr. Percy H. Clarke, J.P., moved and seconded the adoption of the report. After deciding that the name of the hospital should in future be "The Nelson Hospital for Wimbledon, Merton, and District," the numbers of the council of management were increased from twenty-six to thirty-six, thereby including a number of ladies and gentlemen who have taken an active part in the building scheme. The medical and surgical staff were also to be increased from four to six. In this connection it is interesting to note that the medical staff has ceased to receive cases at the old hospital, and those remaining there will be transferred to their new quarters early in Easter week.

### BIRMINGHAM AND THE COAL STRIKE.

The board of management of the Birmingham General Hospital met last Saturday to consider how best to meet the position created by the impending restriction of the gas-supply to what are virtually night hours. Mr. J. B. Clarke presided, and it was decided to appoint a committee. In the meantime, Mr. Howard Collins, the house governor, is reported to be considering how to provide camp cooking apparatus. Circumstances, therefore, have led the committee temporarily to restrict the number of admissions to serious accident cases, and the beds of patients discharged may not be filled with ordinary promptitude.

### A DISPENSARY REPORT CRITICISED.

The report and management of the Leicester People's Dispensary came in for some severe criticism last week. The total expenditure was given as £1,895 odd, which the report stated to be "an increase of £46 odd over the previous year." The deficit on the year's working was given as £124 odd, "resulting partly from the increased payments made to doctors and chemists of the staff." The report further gave generous acknowledgment to the work of the new secretary, Mr. H. Blackwell. A gentleman who was present then rose and characterised the report as "inaccurate and misleading." He challenged the "preponderance of doctors and chemists on the board" and disputed the figures in the report, stating that the expenditure was really £1,867 odd, or a decrease of practically a sovereign, instead of the increase stated, and deposed that £94 odd, and not £124 odd, was the true amount of the deficit. Now these criticisms would seem so easy to test, so manifestly right or wrong, that we note with surprise that it was decided to reconsider them at the next meeting. Accounts are matters that should allow of no misapprehension, and the fact that no one produced evidence, while denying inaccuracy, may create an unfavourable impression in the public mind.

### WHO WILL ADMINISTER SANATORIUM BENEFIT?

The executive of the County Councils Association has decided to submit to the Departmental Committee on Tuberculosis a memorandum urging that the county council should be the local authority to contract with the county insurance committee for the provision of sanatoria benefit. The matter of overlapping, which the present amorphous proposals of the Government's Insurance Act seem likely to produce, is exciting much anxiety among local bodies, and with the vast amount of voluntary money and effort being now spent upon anti-phthisis campaigns, any effort such as that implied in the above memorandum deserves very serious attention.

### THE PROVISION OF SANATORIA

The Birmingham Health Committee has decided to recommend the expenditure of some £30,000 for the extension of the present sanatorium for consumptive cases. This, if the recommendation be adopted, will raise the accommodation from about fifty to one hundred and fifty beds, and the proposal is the outcome of the number of cases reported for which at present no provision has been made. The proposal itself, however, is worth attention, not merely because of the scale on which it is put forward, but as an instance of the diverse ways in which the Government's propositions for the erection or subsidy of sanatoria are being regarded in different parts of the country. From this proposition it might be thought that no State action on a national scale were adumbrated. Elsewhere, however, as instances reported in these columns have shown, a very different attitude is being adopted. Economy is often preached on the ground that the Government's proposals will supply any additional provision which may be needed. Elsewhere again enormous sums of money are being collected from voluntary sources, some of which certainly seem likely to go into the pockets of the builders. Isolation hospitals again are in some cases being converted, and in one or two instances a move has been made on this ground to apply for a Government grant out of the funds which are supposed to be available at the Treasury for anti-phthisis measures. It is really time, in short, that the Government's proposals for dealing with the matter, and for spending whatever money may be available, should be made known. The present risk of overlapping and want of co-ordination is growing serious.

### IMPORTANT HOSPITAL GRANTS IN SERVIA.

According to a Berlin newspaper, proposals for allocating certain sums to hospital construction have been discussed in the present session of the Skupshtina. The proposals include £64,000 for a mental hospital, £49,800 for the completion of the Belgrade public hospital, and £112,000 for the erection of a hospital in the interior of the country.

### HOSPITALS AND THE LATE MR. W. JAMES.

The late Mr. William James, C.V.O., known to the public as an host of the late King, had an interest for hospital workers in that it was he who started with a gift of £10,000 the fund for reconstructing Chichester Infirmary. This fund, it will be recalled, was undertaken as a memorial to King Edward. Mr. James, moreover, was chairman of the Executive Committee of the King Edward VII. Sanatorium at Midhurst. In this connection it may be added that from Mr. James's country house, West Dean Park, King Edward opened an institute for tuberculosis in Montreal by pressing an electric button.

### DISABLED HOSPITAL WORKERS.

The latest report of the Trained Nurses' Annuity Fund for Disabled Nurses states that twenty annuities are now in operation. The Council having previously decided to aim at raising the grants before adding to their numbers it has now become possible to distribute 11s. a week among the annuitants. No annuitant is now receiving less than 10s. a week or its equivalent. The receipts for the year amounted to £822. The claims of this fund are so obvious and the cases often in such distress that there is no need to enlarge upon them here. Dr. Ogier Ward, M.D., is the honorary secretary, and the fund is one which should receive support not only from the public, but from all institutional workers who are in harness and have any donations to spare.

### THE DREADNOUGHT AND ITS SCHOOL.

For the first time for ten years the Seamen's Hospital Society has paid its way. It should be added that the excess of expenditure over income has been occasioned by the enlargement of the Albert Dock Hospital in the year 1900, when the Committee undertook this work in full confidence that in time sufficient support would be forthcoming from the public to justify their action. This has been found to be true, for the income has gradually increased until in the present year there is a surplus of £32. In addition various gifts have been received for endowment which have enabled the Society to return to capital a sum of £6,500, or about one-fifth of the amount withdrawn during the last decade. An attempt has also been made to lighten the report, which shows at a glance the position of the charity, while notes are appended which give in detail the particulars. As regards the schools attached to the Society, the work of the London School of Tropical Medicine has developed to such an extent that it has become necessary again to enlarge the buildings and to provide additional laboratories and accommodation for the residence of students. There are no fewer than sixty-five students in attendance this session. This has strained the resources of the School to the utmost. Our readers will be glad to learn that the meeting held at the Mansion House at the end of February to raise funds for the enlargement of the School has resulted in nearly £4,000 being received so far, but about £10,000 is, as a matter of fact, required.

### KING EDWARD VII. HOSPITAL, WINDSOR.

The first annual meeting of King Edward VII. Hospital, Windsor, since the appointment of Mr. Thomas Carter, the new secretary, took place recently. Prince Christian, the hospital's president, was in the Chair, and alluded to the pathological room which has been recently added, and to the "splendid donation" of £937 which has come from the Aerial Post Fund, of which the Duke of Teck is treasurer. As may have been expected, King Edward's Hospital, Windsor, receives generous support from the Royal family; the King heads the annual subscribers with 50 guineas, and the Prince of Wales has become an annual subscriber, while the Windsor State Apartments Fund has made a further donation of £450. In spite of this assistance, however, the hospital has an adverse balance of £785.

### THE COLLET MEMORIAL AT WORTHING.

The late Dr. Augustus Collet was a man who, as senior medical officer at Worthing Hospital, had earned the respect of many local people. Their desire to connect his name with some visible improvement at the institution, which succeeded in starting a fund for the purpose, was, however, in a sense overshadowed by the proposal to make the new out-patient department a memorial to King Edward. Accordingly Dr. Collet's name could no longer be associated with the extension, but it is satisfactory to learn that a movement is on foot to complete the out-patient department by the installation of *x*-ray and electrical apparatus, and, if possible, to carry the electric lighting from the extension throughout the hospital itself. A Collet memorial on these lines appears to be admirable in intention, and if organised with enthusiasm it should prove successful and extremely popular.

### A BOLD EXTENSION AT BIRMINGHAM.

The committee of the Birmingham and Midland Hospital for Women and the Birmingham Lying-in Charity has decided to enlarge one of its wards by adding eight beds inside and ten outside. One of the causes which has necessitated this enlargement has been the agreement come to a year ago with the health committee, by which cases of puerperal fever were to be dealt with. Indeed, the special work which the hospital has undertaken has proved so costly that there is a balance on the wrong side of the accounts of nearly £3,000. The expenditure necessary for the extension need not be therefore condemned, for if the addition is proved to be necessary by the demands upon the hospital, then the success of an appeal depends merely upon the amount of zeal and ingenuity with which it is prosecuted.

### NOTABLE RE-APPOINTMENTS AT LEICESTER

At a special meeting of the Board of Governors of the Leicester Infirmary, Sir Edward Wood, J.P., has been elected Chairman of the Board for the eleventh year, and Sir Arthur Grey-Hazlerigg, Bart., Deputy-Chairman. The various committees were elected, and Mr. T. Fielding Johnson, appointed Chairman of the House Committee for the seventh year. Dr. Robert Sevestre was elected Hon. Physician to the institution in the place of Dr. F. M. Pope, appointed Consulting Physician. The new Honorary Physician holds the degrees of M.A., M.D., B.C. (Cantab.). He was in January 1899 appointed Assistant Physician to the institution, a post which he has continued to occupy for thirteen years. These re-appointments and elections are notable as showing how this fine institution attaches to itself a group of individuals whose work is a constant one throughout their lives.

### THE HOSTEL OF GOD.

The latest report of the Hostel of God, the free Home for the Dying at 29 North Side, Clapham Common, is an excellent summary of the quite invaluable work which this institution has carried on for twenty years. The need for making the report of a Home of this character attractive is especially necessary, and has been very well recognised by the insertion of two delightful photographs. The first shows the Home as a typical and cheerful house of the Georgian period, with its characteristic white porch, high windows, and creepers; and the second reveals an interior likely to inspire hope and restfulness in the most ill of patients. Owing to a legacy or two, an increased grant from the Sunday Fund, and to the continued help of the Freemasons, a balance on the right side has been recorded, which has been used to increase the endowment fund. Colonel S. P. Jervoise, Chairman of the Council, reminded the public in the general report that the home is strictly free, is really a home, and that admission is gained simply by a medical certificate stating that the applicant is eligible. Dr. M. Mackintosh, the medical officer, gives exceptional praise to the nursing, and goes out of his way to add that "the variety and bounty of the food supplies to patients" have often struck him as being quite unusual in an institution of this kind. The peculiar value of the Home's special work is thus very happily pictured in the different sections of the report.

### AFTER-CARE AND HOSPITAL TREATMENT.

Dr. Nicoll, J.P., who is largely interested in the West Ham branch of the Invalid Children's Aid Society and is vice-chairman of the West Ham and Eastern General Hospital, besides being chairman of the committee of the honorary medical staff, in presenting the annual report last week emphasised particularly the linking up of the various charitable agencies in the borough, such as the hospital, the guardians, the medical officer, the education authorities, and the Charity Organisation Society, which the Children's Aid Society had accomplished. The Duchess of Marlborough appealed for a convalescent home in connection with the preventive work of the Association, notably for pre-phthisic cases, stating that last year the West Ham branch had spent nearly £300 in subscriptions and payments to convalescent homes; and that she thought they could run a suitable home for children for about that sum. Then after Mrs. Munro, who represented the Central Branch, had given some figures of the children helped by the Society, Dr. Couzens, an honorary surgeon at the West Ham Hospital, summed up the practical necessity of such assistance by boldly stating that he had sometimes refused to operate in cases where he could get no guarantee of after-care. This statement alone is enough to make the meeting memorable, for a terse confession of this sort explains the need for after-care with a succinctness that is worth all elaboration. The progress of after-care is very often recorded in these columns, but to whatever lengths it may grow in future, hospital workers will remember with gratitude that the Invalid Children's Aid Society was one of its pioneers, and that both in-patients and out-patients have found in it a tremendous adjunct to hospital treatment.

### AN INTERESTING PROPOSAL AT BOURNEMOUTH.

We have already noted the resignation of Dr. Roberts Thomson from the chairmanship of the amalgamated Royal Victoria and West Hants Hospital at Bournemouth. A proposal is being discussed to commemorate his past services, which included, of course, the difficult task of carrying through the amalgamation. The proposition should excite wide support in Bournemouth, which has known Dr. Roberts Thomson for so many years, and if it matures we hope that it may take the form most likely to benefit the hospital. The Royal Victoria and West Hants Hospital has, no doubt, to contend against a certain conservatism on the part of the public in the matter of subscriptions, and this cannot be expected to die away at once. Affection for one or other of the former constituents of the present institution probably will influence subscribers for a year or two, but now that the re-formed institution has begun its new career all the support which it can secure is advisable, and no better motive could be found than a desire to do honour to the memory of the ex-chairman.

### THE SECOND COTTAGE HOSPITAL IN ENGLAND.

In a recent article in these columns we took occasion to set side by side the work of Lord Lister with that of a great general practitioner since the funerals of the two took place contemporaneously. The late Dr. J. B. Maurice, the great "G.P.," devoted much of his vast energy towards the building up of Savernake Cottage Hospital, and at the annual meeting the story of its development was heard from the lips of its founder, the Rev. J. O. Stephens, who journeyed from London to preside on this occasion. From 1861 to 1879 Mr. Stephens was vicar of Savernake, and in 1866 the hospital, which has the honour of being the second cottage hospital in England, was established in an abandoned laundry, which the then Lady Ailesbury gave for the purpose. In 1872 the new hospital was built, and as it was Mr. Stephens' and the late Dr. Maurice's intention to make it self-supporting in the district, this new building was the concrete proof of the success of Mr. Stephens' peripatetics through 45 villages, in practically all of which the clergy promised to devote one Sunday's offering to the support of the hospital. It was never, therefore, dependent upon the Ailesbury family, and during the thirteen years Mr. Stephens was responsible for its management there was only a deficit once, which was paid off by a special effort the following week. Of the four trustees who were originally appointed Mr. Stephens is the only survivor, and the success of Savernake Cottage Hospital, which we emphasised in the article above referred to, shows how little grounds there were for the objection against which Mr. Stephens had to contend most strenuously—that whether a cottage hospital were established or not, in any event all serious cases must be sent to London. The number of important operations yearly performed at Savernake Cottage Hospital has now risen to 200. It is a fine reward for the founder of the second cottage hospital in England to have lived to see the objections of the seventies thus nobly put to the disproof.

### THE WORK OF THE INFANTS' HOSPITAL.

The report of the Infants' Hospital, Vincent Square, is always one of those to which we turn with pleasure. This year its attractiveness of appearance is maintained, and we note that the outpatient department is extending its activity in giving advice in the case of infants who are unsuitable for admission to the wards, and in continuing the treatment of certain infants after their discharge. This, no doubt, will prove a growing and admirable branch of the work. The secretary, Mr. Alfred G. Small, is to be congratulated on the sixty new annual subscriptions which were obtained in the course of the year. The research fund received £500 from Mr. Emile Merton, and the laboratory, which has owed from the first so much to the treasurer, Mr. Robert Mond, has extended its scope so greatly that a research committee has been appointed to govern its administration. Perhaps it is advisable to add that the laboratory is maintained only by funds left for this specific purpose. The milk supply is maintained on the lines we have previously noted, and continues to prove satisfactory. Such a report should certainly consolidate the support of the subscribers.

### DISPENSARY LIFE IN INDIA.

Seven years ago or thereabouts Dr. M. I. Philip, a practitioner in India, gave up practice in the town of Aleppev, and went at the bidding of the chief medical officer of State to Komerom to start medical work for the relief of its plague-stricken and destitute population. He was assisted by the Government of Travancore with an annual grant. No sooner, however, had the dispensary which he had founded been established in the affections of the inhabitants than this grant was withdrawn, leaving Dr. Philip saddled with the institution which his energy had founded, at the very time when he was without personal resources from the private practice in which he was no longer engaged. Such, in brief, is the depressing story which has reached us from Dr. Philip, authenticated by divers testimonials, by a legal opinion on the rights of his case, by medical and ecclesiastical affirmations, and by certain leading articles translated from the vernacular newspapers. The energy in the face of keen disappointment which is displayed by the writer in sending an appeal to London, in the hope that voluntary charity may help to save him and his dispensary, is remarkable enough, and the very remoteness of the institution and of the interests concerned give it a special interest for the hospital world at home in England. We are apt to think that institutional life means life in an institution in England, that hospital appeals mean appeals to English people, and suddenly, in the midst of our routine work, there falls upon us this echo from an institution so small and so distant that it takes time to realise that it actually has to fight for its existence in the same way as, though in an intenser degree than, that in which the fight is carried on in London. The acute contrast which this appeal provides demands attention. The Anglican Bishop of Travancore and Cochin, India, will receive any help that our readers may care to send.

### UNIVERSITY COLLEGE HOSPITAL.

Popularly considered, the most novel element in the latest report of University College, London, is the record of the new department for cardiography, which has been established under the charge of Dr. Thomas Lewis. The report also reminds the public that its most famous medical student, Lord Lister, has left an important legacy to the hospital. Captain the Hon. E. S. Dawson presided at the annual meeting, and he and the recently appointed honorary treasurer, Sir Ernest Hatch, exchanged views on the probable effect of the Insurance Act. Captain Dawson took a pessimistic view, especially as regards the hospital's hope of future legacy, whereas Sir Ernest Hatch believed that its disastrous effects would prove to be only temporary. Thanks to the action of the trustees of the Annie Zunz fund, Ward 15 has been opened, whereby the number of inpatients during the past two years has increased by 851.

### TO OUR READERS.

Contributions are specially invited from any of our readers to these columns. They should deal with topical subjects and news. They must be authenticated for the information of the Editor only. The minimum payment if published is 5s. There is no hard-and-fast rule as to space, but notes of about twenty lines in length are preferred.

